# Natural Killer Cell Function and Dysfunction in Hepatitis C Virus Infection

**DOI:** 10.1155/2014/903764

**Published:** 2014-06-25

**Authors:** Kayla A. Holder, Rodney S. Russell, Michael D. Grant

**Affiliations:** Immunology and Infectious Diseases Program, Division of BioMedical Sciences, Faculty of Medicine, Memorial University of Newfoundland, 300 Prince Phillip Drive, St. John's, NL, Canada A1B 3V6

## Abstract

Viruses must continually adapt against dynamic innate and adaptive responses of the host immune system to establish chronic infection. Only a small minority (~20%) of those exposed to hepatitis C virus (HCV) spontaneously clear infection, leaving approximately 200 million people worldwide chronically infected with HCV. A number of recent research studies suggest that establishment and maintenance of chronic HCV infection involve natural killer (NK) cell dysfunction. This relationship is illustrated *in vitro* by disruption of typical NK cell responses including both cell-mediated cytotoxicity and cytokine production. Expression of a number of activating NK cell receptors *in vivo* is also affected in chronic HCV infection. Thus, direct *in vivo* and *in vitro* evidence of compromised NK function in chronic HCV infection in conjunction with significant epidemiological associations between the outcome of HCV infection and certain combinations of NK cell regulatory receptor and class I human histocompatibility linked antigen (HLA) genotypes indicate that NK cells are important in the immune response against HCV infection. In this review, we highlight evidence suggesting that selective impairment of NK cell activity is related to establishment of chronic HCV infection.

## 1. Host Invasion and Immune Evasion

Human immunity is classically divided into innate and adaptive components. The adaptive immune response is generally regarded as being uniquely mediated by B and T lymphocytes, as it is only progenitors of these cells that undergo somatic recombination-activating gene- (Rag-) dependent variable (V) gene rearrangement in order to produce a diverse clonotypic repertoire of antigen-specific receptors [1]. Antigen-mediated clonal selection, leading to expansion and persistence of particular cells or their products at elevated levels, provides the adaptive immune system with specificity and memory. In contrast, innate immune responses offer a first line of defense, stemming from cells and mechanisms that recognize pathogen-associated molecular patterns (PAMPs) in a generic, nonspecific, and noninstructive manner [2]. Coexistence of persistent viruses and their hosts exerts selective pressures on both the host immune system and on viral genomes, forcing viruses to continually evolve mechanisms through which host immune defenses are evaded.

Viral evasion strategies can include antigenic variation, synthesis of decoy proteins that inactivate immune responses, production of proteins (immunoevasins) that compromise antigen presentation, and production or induction of proteins that disrupt host humoral and cellular immune responses and/or effector functions [2, 3]. While T-cell-mediated immune responses provide long-term control of viral infections, initial management of these infections by natural killer (NK) cells, prior to development of the adaptive immune response, is thought to be crucial. In humans, depressed NK cell function is associated with sensitivity to viral infections [4]. Of particular note, Biron et al. described the case of a patient with genetic NK cell deficiency and extreme sensitivity to herpes virus infections, despite having normal numbers of B and T lymphocytes [5]. Multiple NK cell studies in the context of viral infection indicate that viruses evade immune pressure by generating variants that modulate recognition of infected cells by NK cells. Furthermore, NK cells are not only important for direct early control of viral infections, but they also contribute to induction of the adaptive antiviral immune response by releasing immunomodulatory cytokines and chemokines [6] and through bidirectional interactions with dendritic cells (DC) (reviewed in [7, 8]). These reciprocal interactions ultimately drive the T-cell immune response and, in some cases, culminate in reduced viral replication or even clearance of viral infection [9]. Recent studies also demonstrate that murine and possibly human NK cells have receptors specific for cytomegalovirus (CMV) that enable selective proliferation and expansion of NK subsets, thus endowing NK cells with limited properties previously attributed exclusively to T and B lymphocytes [10–13].

Epidemiological studies suggest that NK cells play a role in determining the outcome of hepatitis C virus (HCV) infection [14, 15]. Here, we will consider the effects HCV infection has upon NK cells by reviewing the epidemiological associations, noting* in vivo* evidence of NK cell dysfunction in chronic HCV infection and discussing recent* in vitro* experiments indicating that direct interaction between circulating NK cells and HCV-infected cells impairs NK cell function.

## 2. Hepatitis C Virus

Approximately 3% of the world's population is infected with HCV [16], an enveloped, positive-sense RNA virus of the* Hepacivirus* genus within the Flaviviridae family [3]. The HCV RNA genome is encased by core protein multimers to form the viral nucleocapsid that is surrounded by an endoplasmic reticulum (ER) membrane-derived envelope studded with HCV envelope proteins 1 and 2 (E1/E2) [17, 18]. Host cell infection with HCV occurs through the interaction of HCV E1 and/or E2 with multiple cellular coreceptors including CD81 (also termed target of antiproliferative antibody 1 (TAPA1)) [19–23], scavenger receptor class B type I (SRBI) [24–26], occludin (OCLN) [27–29], and claudin-1 (CLDN1) [30, 31]. In the absence of effective treatment, approximately 80% of individuals infected with HCV fail to mount an immune response adequate for viral clearance and, consequently, develop chronic infection and suffer an increased risk for liver fibrosis and hepatocellular carcinoma [32–34]. While approximately 20% of HCV-infected individuals spontaneously clear infection, the mechanism of spontaneous clearance remains poorly defined and a greater understanding of both the viral clearance process and of viral strategies underlying immune escape is necessary for future vaccine development and more effective management of infection. There is mounting evidence that NK cells are involved in the clearance and control of HCV infection, including associations between NK cell dysfunction, chronic HCV infection, and the pathogenesis of liver disease [14, 35, 36].

**Figure 1:**
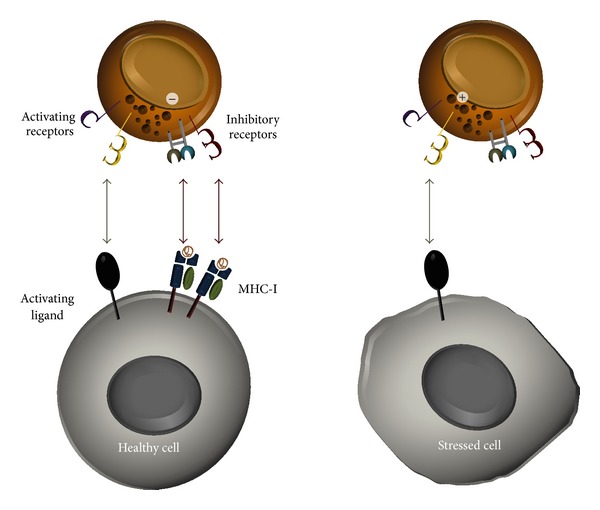
Natural killer cell natural cytotoxicity. NK cell inhibitory receptors recognize “self” MHC-I molecules which can prevent cytolysis of healthy cells even when activating receptors interact with their ligands. Transformed or virus-infected cells often reduce MHC-I expression; thus when a NK cell activating receptor engages its ligand, there is no negative signal to overcome the positive activating signal and the target cell is destroyed through a perforin and granzyme apoptosis-inducing mechanism.

Natural killer cells are lymphocytes that develop in the bone marrow, differentiate in lymphoid tissues, and migrate into various tissues [37]. The environment in which a NK cell matures impacts NK cell phenotype and function giving rise to a heterogeneous population. Although NK cells can be prominent in nonlymphoid tissues such as the liver and decidua [38–40], the functions of these NK cells can differ greatly from the functions of those in peripheral blood. The NK cells that comprise between 5 and 20% of peripheral blood lymphocytes provide inherent defense against some transformed cells and a variety of pathogens, including viruses [5, 41–43]. First identified in 1975 and classically defined as cytolytic lymphocytes, NK cells remain categorized as innate immune effector cells since they do not undergo receptor gene rearrangement [41, 44]. Thus, selective effector function against infected or transformed cells is mediated by composite integration of signals received through a diverse assortment of germ-line encoded activating and inhibitory receptors interacting with their respective ligands [45] (Figure 1).

## 3. Natural Killer Cell Functions

Peripheral NK cells primarily fall into two major functionally distinct subsets. The highly cytotoxic “mature” CD56^dim⁡^CD16^bright^ NK cells are more responsive to cell surface ligands than the “immature” CD56^bright^CD16^dim⁡^ NK cells, which are more responsive to soluble factors [6, 46]. The CD56^bright^CD16^dim⁡^ NK cells are less cytotoxic but predominantly secrete cytokines, such as proinflammatory interferon- (IFN-) *γ* and tumour necrosis factor- (TNF-) *α*, or immunoregulatory interleukin- (IL-) 10 [47, 48].

**Figure 2:**
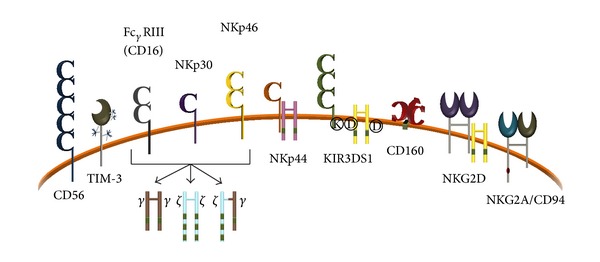
Natural killer cell receptor repertoire. Natural killer cells express some fraction of a diverse set of germ-line encoded receptors, many of which display allelic polymorphism. They are variously associated with different adaptor molecules that convey either activating or inhibitory signals. Two of the three natural cytotoxicity receptors, NKp30, NKp46, and also the CD16 Fc_*γ*_RIII receptor, mediate signaling through ITAM-containing *ζ* chain homodimers, Fc_*ε*_RI_*γ*_ homodimers, or Fc_*ε*_RI_*γ*/*ζ*_ heterodimers. Activating receptors depicted here are CD16, NKp30, NKp46, NKp44, KIR3DS1, CD160, and NKG2D; inhibitory receptors depicted are TIM-3 and NKG2A/CD94. The CD56 molecule is comprised of five immunoglobulin-like domains and, in the absence of CD3, is a reliable marker for NK cells.

**Figure 3:**
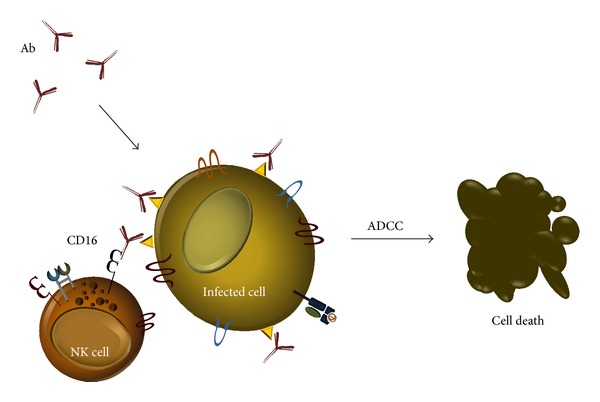
Natural killer cell antibody-dependent cell-mediated cytotoxicity (ADCC). Natural killer cells can recognize and kill antibody-coated target cells. When antibodies bind antigens displayed on the surface of transformed or infected target cells, NK CD16 receptors bind the Fc portion of bound antibodies (most IgG subclasses) and mediate cytotoxicity against the target cell by degranulation and directed release of cytotoxins.

Through their constellation of cell surface receptors, NK cells can detect variations in cell surface composition manifest through a multitude of ligands and integrate this information to determine the type and intensity of response that is appropriate [49]. With no prior exposure, NK cells can recognize and kill target cells that have downregulated expression of class I human histocompatibility-linked leukocyte antigen- (HLA-) I molecules, a process that occurs during many viral infections [45]. In humans, the NK receptors that recognize class I HLA molecules, predominantly killer cell immunoglobulin-like receptors (KIR), can convey NK cell activating signals through short (S) cytoplasmic domains or NK cell inhibitory signals if the KIR possesses a long (L) cytoplasmic domain [50] (see Figure 3). Inhibitory NKG2A receptors can also prevent cytolysis of healthy cells by recognizing expression of nonclassical class I HLA-E complexes [45, 51–53]. As per the “missing self” hypothesis, healthy cells expressing normal amounts of class I HLA complexes are less sensitive to NK cell lysis than are transformed or virus-infected cells with decreased HLA-I expression [54] (Figure 2). Virus-infected cells expressing adequate amounts of class I HLA molecules generally remain protected from NK cell-mediated lysis. Therefore, maintaining expression of class I HLA molecules, especially HLA-E, is one mechanism by which pathogens can thwart NK cell surveillance.

## 4. Natural Killer Cells in HCV Infection

Natural history studies have revealed that HCV-exposed individuals with coordinate expression of certain KIR variants and their corresponding HLA-I ligand gain a higher probability of spontaneous HCV clearance than those without [14, 55–58]. To maintain functional recognition of rapidly evolving class I HLA complexes, the NK cell KIR genes must also evolve under pathogen-mediated pressure [59, 60]. Genotyping of those exposed to HCV demonstrated that coordinate expression of NK cell receptor KIR2DL3 and its cognate class I HLA C group 1 (HLA-C1) ligand confers an increased likelihood of spontaneous HCV clearance or of a sustained virological response (SVR) to treatment when spontaneous HCV clearance is not achieved [14, 56, 61]. One functional interpretation of this association is that as the interaction between KIR2DL3 and HLA-C1 is relatively low affinity, it generates a weaker inhibitory signal than other KIR/ligand interactions allowing a greater functional response by NK cells [57, 58]. Other KIRs that have been identified as relevant to the outcome of HCV infection and treatment efficacy are 2DL2, 2DL3, 2DS1, 2DS2, and 3DL1 [61, 62]. The expression of KIR3DL1 is decreased in individuals with HCV infection, suggesting that this receptor may also be involved in the regulation of chronic HCV infection [55]. Expression of KIRs with short cytoplasmic tails promotes target cell cytolysis and IFN-*γ* production through DAP12 signaling (see Figure 2) upon recognition of cognate class I HLA ligands [50, 63, 64]. Although the precise nature of its HLA ligand has yet to be identified, the activating receptor KIR3DS1 appears to support protection against hepatocellular carcinoma in those chronically infected with HCV and favors HCV genotype 1a clearance in response to combination treatment with ribavirin and pegylated IFN-*α* [63, 65]. Favourable genetic combinations of KIR and class I HLA complexes contribute to the NK cell response not only in acute HCV infection, but also towards controlling disease progression in those chronically infected and towards sustained virological responses in those receiving treatment. Viruses can evade the NK cell immune response through the generation of variants that can escape stimulatory and/or enhance inhibitory NK cell receptor (NKR) recognition.

Some studies indicate that the HLA-E molecule, which is a ligand for the NK inhibitory receptor NKG2A, is upregulated on hepatocytes in complex with an HCV core protein peptide (aa35–44) during chronic HCV infection [66]. Nattermann et al. reported an increased expression of NKG2A/CD94 inhibitory receptors on circulating NK cells in patients with chronic HCV infection; however, in an* in vitro* model representative of acute HCV infection, neither NKG2A nor HLA-E expression increased on NK or HCV-infected cells, respectively [67–69]. Nonetheless, to combat NK cell-mediated control of infection, HCV may employ multiple means of increasing expression of natural or decoy ligands for inhibitory NKRs as chronic infection develops and disease progresses.

Since viral infection often results in reduced cell surface class I HLA expression, NK cell inhibitory signals will be dampened, allowing NK cells to lyse altered autologous target cells if activating receptors such as the natural cytotoxicity receptors (NCRs) NKp46, NKp44, NKp30, or NKG2D or nonclassical class I HLA-E-specific NCRs CD94/NKG2C and CD94/NKG2E are engaged (see Figure 2) [45, 70–75]. In addition, CD16 (Fc_*γ*_RIII) is a low-affinity activating receptor specific for the constant (Fc) region of immunoglobulin G (IgG) that triggers antibody-dependent cell-mediated cytotoxicity (ADCC) upon recognition of antibody-coated cells (Figure 3) [45]. The only NCR ubiquitously expressed on NK cells is NKp46, and while NKp30 and NKp46 are constitutively expressed on subsets of NK cells, NCR NKp44 expression is restricted to activated NK cells [72, 76]. Many ligands of viral origin have been identified for NK activating receptors, which can serve as decoys and disrupt positive NK cell stimuli. Of note, human (H)CMV capsid protein pp65 disrupts NKp30 expression and influenza hemagglutinin (HA) can interact with, and inhibit, NK cells through NKp30, NKp44, and NKp46 [77–79]. Although no NCR ligands resulting from HCV infection have yet been identified, studies have reported decreased NK cell activating receptor expression on NK cells from chronically infected individuals. A progressive increase in the proportion of NKG2C^+^ NK cells was reported to occur following liver transplantation for chronic hepatitis C and was initially thought to be associated with HCV recurrence [80]. A similar NK subset observed in human immunodeficiency virus infection expressed reduced levels of the NCRs NKp30, NKp44, and NKp46 [81]. Expansion of this CD57^+^NKG2C^+^ NK subset has since been shown to relate primarily to CMV infection and involve selection of NK cells expressing KIR capable of licensing NK effector function and enforcing self-tolerance [82, 83]. Despite evidence for downregulation of NCRs on this NK subset, they are polyfunctional in terms of cytokine expression and demonstrate enhanced cytotoxicity against antibody-coated target cells [82, 84].

Freshly isolated peripheral blood and liver-derived NK cells from chronically infected HCV patients exhibited significantly lower levels of NCR NKp30 and NKp46 expression than the NK cells of healthy controls [67]. Although no differences in NKG2C or NKG2D surface expression were noted, some* in vitro* experimental data suggested downregulated NKG2D and/or NKp30 surface expression after* ex vivo* exposure of NK cells to HCV-infected cells [68, 69]. Direct contact between NK cells and HCV-infected cells* in vitro* promotes downregulation of NKp30, suggesting that HCV can affect NK cell recognition and function through upregulation of an as yet unidentified ligand that physically interacts with NKp30 [69]. This possibility is supported by the demonstration of increased binding of recombinant NKp30 protein to HCV-infected Huh-7.5 cells [69]. Cytotoxicity and IFN-*γ* production by NK cells are also reduced following short (5 hours) or extended (18 hours) exposure to HCV-infected Huh-7.5 cells [67–69, 85, 86].

Both NKR expression itself and/or variation in NKR ligand expression can impact NK cell functions. The T-cell immunoglobulin mucin-3 (TIM-3) receptor was recently shown to inhibit cytotoxic and cytokine responses of NK cells upon interaction with galectin-9 (Gal-9) or phosphatidylserine (PtdSer) on target cells [87–92]. As Gal-9 binding depends on recognition of certain carbohydrates and the extent of NK cell receptor ectodomain glycosylation can vary, the degree of ligand recognition and binding may also vary, thereby providing another level of regulatory control over NK cell functions [93–95]. Circulating levels of the Gal-9 ligand for NK cell TIM-3 receptors are significantly increased in HCV-infected individuals compared to uninfected controls [96]. As this interaction mediates an inhibitory response, this is another potential mechanism through which HCV can inhibit typical NK functions to favor establishment of persistent infection. While many studies have investigated the suppressive effect of TIM-3 in the context of effector T-cell functions on HCV infection, the impact of TIM-3 on NK cells with respect to HCV infection has not yet been extensively studied [84, 85].

Several previous studies investigating the role of NK cells in HCV infection focused on interactions between HCV E2 and CD81 on NK cells as a potential mechanism underlying NK cell dysfunction [97–99]. These studies exposed IL-2-stimulated or purified NK cells to soluble and immobilized recombinant HCV E2, anti-CD81 monoclonal antibodies, or plate-bound cell culture-derived HCV (HCVcc) [97, 98]. Engaging NK CD81 in these ways decreased IFN-*γ* production and NK cell cytotoxicity; therefore, cross-linking of NK CD81 by HCV E2 has been proposed as an NK inhibitory mechanism promoting HCV persistence [100–103]. However, more recent studies reported no significant change in NK cell cytotoxicity when NK cells were exposed to intact, infectious cell-free HCVcc at supraphysiological levels [69, 99]. These results suggest that although HCV E2 may specifically bind to CD81 on NK cells, the configuration of E2 within an intact infectious virus particle does not facilitate the degree of NK CD81 receptor cross-linking necessary to mediate an inhibitory signal [97, 98]. Most of these systems used peripheral NK cells stimulated* in vitro* with recombinant IL-2 (rIL-2), rIL-12, or IFN-*α* combined with extended coculture with HCV-infected cells, introducing additional complications to interpreting* in vivo* relevance of the reported effects [67, 68, 97, 98, 104].

One recent study used the monoclonal antibody AP33, a broadly neutralizing antibody against the CD81-binding site on HCV E2 (amino acid residues 412–423) [105–107], in conjunction with HCV-infected human hepatoma (Huh-7.5) and unadulterated NK cells to prevent E2/CD81 interactions and probe the significance of NK CD81/HCV E2 interactions in the context of NK cell inhibition. Firstly, there was no evidence of cell surface expression of HCV E2 on HCV-infected Huh-7.5 cells, and secondly, blocking potential HCV E2/NK CD81 interactions with either AP33 or soluble anti-CD81 had no significant effect on NK cell inhibition mediated by HCV-infected cells. These data provide compelling evidence that an HCV E2/NK CD81 interaction does not underlie the inhibition of NK cytotoxicity mediated by HCV-infected cells* in vitro* [69, 99].

Chronic viral infection requires successful evasion of innate and adaptive host immune responses. Through interaction with host innate and adaptive immune responses, HCV has evolved mechanisms to circumvent immune pressures and to establish chronic infection in the majority of cases. Numerous experimental studies demonstrate NK cell dysfunction in HCV infection and epidemiological data suggests that NK cells play some role in clearance or control of HCV infection [14, 35, 36, 69]. Since NK cells uniquely bridge innate and adaptive immune responses, their sufficient function may be especially important in initial and lasting control of HCV infection.
